# Health Care Contact Days for Older Adults Enrolled in Cancer Clinical Trials

**DOI:** 10.1001/jamanetworkopen.2025.0778

**Published:** 2025-03-13

**Authors:** Arjun Gupta, Cathee Till, Riha Vaidya, Dawn L. Hershman, Joseph M. Unger

**Affiliations:** 1 Division of Hematology, Oncology, and Transplantation, University of Minnesota, Minneapolis; 2 SWOG Statistics and Data Management Center, Fred Hutchinson Cancer Center, Seattle, Washington; 3 Now with Flatiron Health Inc, New York, New York; 4 Department of Medicine, Columbia University, New York, New York

## Abstract

**Importance:**

Contact days—days with health care contact outside the home—are a measure of how much of a patient’s life is consumed by health care. Clinical trials, with a more uniform patient mix and protocolized care, provide a unique opportunity to assess whether burdens differ by individuals’ sociodemographic backgrounds.

**Objective:**

To characterize patterns of contact days for older adults with cancer participating in clinical trials.

**Design, Setting, and Participants:**

In this cohort study, data from 6 SWOG Cancer Research Network trials across prostate, lung, and pancreatic cancers that recruited patients aged 65 years or older from 1999 to 2014 were linked with Medicare claims data. Data were analyzed from December 14, 2023, to September 26, 2024.

**Exposures:**

Demographic variables, including age, sex, self-reported race and ethnicity, and insurance status; clinical factors, such as cancer type and study-specific prognostic risk score; and social factors, such as neighborhood socioeconomic deprivation.

**Main Outcomes and Measures:**

Number of contact days, defined as number of days with contact with the health care system, percentage of health care contact days (number of contact days divided by follow-up), and sources of contact days (eg, ambulatory or inpatient) in the first 12 months after trial enrollment. Sociodemographic and clinical factors associated with contact days were examined using negative binomial regression, including an offset variable for duration of observation.

**Results:**

The study included 1429 patients (median age, 71 years [range, 65-91 years]; 1123 men [78.6%]; and 332 patients [23.5%] with rural residence). The median number of contact days was 48 (IQR, 26-71), of a median of 350 days (IQR, 178-365 days) of observation; the median percentage of contact days was 19% (IQR, 13%-29%). The most common sources of contact days were ambulatory clinician visits (median, 17 [IQR, 7-25]), tests (median, 12 [IQR, 3-24]), and treatments (median, 11 [IQR, 3-22]). A median of 70% (IQR, 50%-88%) of ambulatory contact days had only a single service performed on that day (eg, only tests). In multivariable regression, factors associated with increased contact days included age (relative risk [RR] per year, 1.02 [95% CI, 1.01-1.02]), insurance type (Medicare alone or with Medicaid or private insurance vs other: RR, 2.47 [95% CI, 2.16-2.83]), prognostic risk score (above the median vs at or below the median: RR, 1.14 [95% CI, 1.04-1.25]), and type of cancer (pancreatic vs prostate cancer: RR, 1.69 [95% CI, 1.51-1.89]; lung vs prostate cancer: RR, 1.69 [95% CI, 1.54-1.85]).

**Conclusions and Relevance:**

In this cohort study of older adults with advanced stage cancer participating in phase 3 randomized clinical trials, patients spent nearly 1 in 5 days with health care contact. These findings highlight the need to simplify trial requirements to minimize participant burden.

## Key Points

**Question:**

What are the patterns of health care contact days among older adults with cancer participating in clinical trials?

**Findings:**

In this cohort study of 1429 older trial participants, the median percentage of days with health care contact was 19%, and 70% of ambulatory contact days had only a single service performed (eg, only tests). Factors associated with increased contact days included age, insurance type, prognostic risk, and type of cancer but not rural residence or neighborhood deprivation.

**Meaning:**

These findings underscore the need to simplify trial requirements to minimize participant burden while increasing confidence that participants from diverse geographic areas receive equitable care in trials.

## Teaser

This cohort study uses data from 6 SWOG Cancer Research Network trials to examine patterns of health care contact days among adults aged 65 years or older with cancer participating in clinical trials.

## Introduction

Health care contact days—days with health care contact outside the home—are a measure of how much of a patient’s time is consumed by health care.^1,2,3,4^ This number of contact days is especially relevant for older adults with advanced cancer facing limited survival.^5,6,7,8^ Previous studies have demonstrated that patients with advanced gastrointestinal cancer and lung cancer in clinical practice outside of clinical trials have a median survival in the range of several months, and approximately 25% to 33% of these days are spent with health care contact.^6,8,9,10^ Rates of contact days are similar for early-phase clinical trial participants.^11,12^ These similar rates may be explained by the fact that, while patients treated in routine practice tend to be older, with more comorbidities and poorer physical function, resulting in more unplanned contact days, early-phase clinical trials often impose significant protocol-related needs, resulting in more planned contact days.^5^ Patterns of contact days for participants in later-phase trials have not yet been well described but are important for characterizing the potential burden of trial participation.

Alongside a measure of burden, contact days also represent access to often necessary care.^13^ Prior work has shown that contact days vary not just by clinical factors but also by sociodemographic factors, such as rural vs urban residence.^4,9,10^ These studies are limited by the significant heterogeneity in clinical needs, treatments, and care delivery across patients and health systems, making it difficult to draw definitive conclusions regarding these associations. Concern remains that patients with different sociodemographic backgrounds may experience more contact days due to uncoordinated care or fewer contact days on account of barriers in accessing care. Thus, there is a need to understand the factors associated with contact days in clinical trials. Later-phase clinical trials represent the ideal avenue to study this because (1) inclusion criteria ensure a level of clinical homogeneity, (2) participants tend to receive more protocol-guided care, and (3) protocol-mandated “extra” visits are limited, relative to early-phase trials.^5^ In this study, we sought to characterize the rates and patterns of contact days experienced by older adults with advanced cancer enrolled in randomized clinical trials and identify factors associated with contact days.

## Methods

This cohort study followed the Strengthening the Reporting of Observational Studies in Epidemiology (STROBE) reporting guideline. Informed consent of study participants for this cohort study was not required because secondary data that were not identifiable were used. Approval to conduct this research was obtained from the Fred Hutchinson Cancer Center institutional review board. We included data from SWOG Cancer Research Network trials for patients with prostate (trial numbers S9916 and S0421), lung (trial numbers S0003, S0124, and S0819), and pancreatic (trial number S0205) cancers. We selected these trials because they enrolled patients with advanced cancer (when patients may face limited survival and experience high rates of health care contact), were linked to Medicare claims (linkage is through Social Security numbers, which is optional), and had a reasonable proportion of older adult participants (for whom Medicare claims are applicable). These trials recruited participants from 1999 to 2014. Trial records were linked to Medicare claims data by Social Security number, sex, and date of birth. To be included, patients were required to be aged 65 years or older at enrollment and to have continuous Medicare Parts A and B coverage immediately after baseline, until 12 months or death, whichever comes first, with no concurrent health maintenance organization coverage.

Demographic variables, including age, sex, self-reported race (Black, White, other [which includes Asian, American Indian or Alaska Native, Pacific Islander, multiracial, and unknown]), self-reported ethnicity (Hispanic and non-Hispanic), and insurance status, were obtained at enrollment. Race and ethnicity were assessed in this study because these variables might be expected to be associated with contact days. Potential differences in prognostic risk across the different studies were accounted for using a study-specific prognostic risk score. For each study, we identified the key baseline clinical risk factors that were included as stratification variables. We then summed the number of adverse clinical risk factors, creating a composite risk score, and defined *high risk* as having a risk score above the median for each study (eTable 1 in Supplement 1). Approximate 1-way travel distance was calculated as the number of kilometers between a patient’s residence zip code and the zip code of the patient’s study site.

Neighborhood socioeconomic deprivation was measured using patients’ residential zip codes in 2 ways. First, the zip code was linked to the area deprivation index (ADI), measured on a scale of 0 to 100, with a higher ADI score denoting areas of higher deprivation. The ADI score was split into tertiles based on overall US distribution of the ADI. Second, rural or urban residency was defined using the US Department of Agriculture Economic Research Service rural-urban continuum codes (1-3: urban; 4-9: rural). Patients missing a zip code were not included in geography-related analyses.

Health care contact days, defined as the number of days with contact with the health care system, were calculated using MedPAR, Outpatient, Hospice, and Carrier files (eTable 2 in Supplement 1). The MedPAR file includes inpatient and skilled nursing facility (SNF) claims, and the Carrier file includes physician claims. Days of contact were categorized as inpatient care (hospital stay, emergency department visit, SNF stay, hospice care, and observation stay) and ambulatory care (clinician visits, tests such as bloodwork, imaging studies such as ultrasonography, procedures such as endoscopy, and treatments such as infusions). Patients with more than 1 category of health care contact on a single day were placed in a single category using the following hierarchy: hospital stay, then emergency department visit, then SNF stay, then hospice care, then observation stay, and then ambulatory care. Patients with more than 1 category of ambulatory care (and no inpatient care on that day) were placed in all relevant ambulatory care categories, with only 1 day added to total contact days.

Patients reporting private insurance or Veterans Affairs coverage while also linked to Medicare may have had missing health care utilization data, if the non-Medicare payer covered the cost of treatment rather than Medicare. Therefore, patients with no observed health care utilization were excluded, as their health care utilization data were considered to be missing rather than reflecting zero health care utilization, unless they had a protocol deviation recorded, which could suggest, instead, zero health care utilization that was expected rather than missing data. We reviewed trial protocols and summarized the expected number of protocol-mandated contact days.

### Statistical Analysis

We analyzed data from December 14, 2023, to September 26, 2024. To evaluate whether there was any bias in the included sample, baseline characteristics were compared between the patients included in this analysis and the patients (also aged ≥65 years) who were not included, using χ^2^ tests. Summary statistics of health care utilization are presented overall and stratified by study. To understand ambulatory care coordination, we calculated the percentage of ambulatory contact days with only 1 service performed (eg, only tests or only a clinician visit) and the percentage of ambulatory days with a clinician visit performed on the same day as another ambulatory service.

To account for overdispersion of the observed data, negative binomial regression, with a log link and clustering by study ID, was used to assess the association between variables and contact days. Because total time under observation varied due to patient deaths, an offset variable for duration of observation was included. The demographic variables considered included age (continuous), self-reported race (Black, White, and other [which includes Asian, American Indian or Alaska Native, Pacific Islander, multiracial, and unknown]), self-reported ethnicity (Hispanic or non-Hispanic), sex (male or female), insurance status (Medicare alone or with private insurance or other), geographic location (urban or rural), and ADI tertiles. Clinical variables included prognostic risk score (at or below median or above the median), type of cancer (pancreatic, prostate, or lung), and performance status at enrollment (0-1 or ≥2). Variables were examined individually in univariate analysis, as well as in multivariate analysis mutually adjusted for each other. Exponentiated coefficient estimates, reported as relative risk (RR), and 95% CIs are reported.

A sensitivity analysis was performed, excluding patients with no observed health care utilization, regardless of protocol deviation. An additional sensitivity analysis, examining only inpatient contact days as an outcome to measure unplanned contact, was also performed.

To understand the trajectory of contact days over the trial and disease course, we divided the follow-up time for each participant into 20 equal time periods and calculated the percentage of contact days during each period. We plotted the contact day rate (y-axis) with the percentage of time elapsed (x-axis) and fit a cubic smoothing spline to the normalized observations. Because some patients were alive beyond 1 year, we stratified the trajectory-based analysis by whether the patient’s complete trajectory was represented (died within 1 year) or not (alive >1 year).

All analyses were conducted in SAS, version 9.4 (SAS Institute Inc) and R, version 3.6.1 (R Project for Statistical Computing). A 2-sided *P* < .05 was considered statistically significant.

## Results

After exclusions, a total of 1429 patients were included (median age, 71 years [range, 65-91 years]; 332 patients [23.5%] with rural residence) (eTable 3 in Supplement 1). The study included 1123 men (78.6%) and 306 women (21.4%), 109 Black patients (7.6%), 38 Hispanic patients (2.7%), 1280 White patients (89.6%), and 40 patients of other or unknown race and ethnicity (2.8%). Table 1 presents detailed patient characteristics. Compared with patients aged 65 years or older not included in the analysis, the patients included were more likely to live in a rural zip code area or an area of higher deprivation, to have private health insurance, and to be in a lung or prostate cancer study.

**Table 1.** Summary Statistics of Participant Characteristics at Baseline

Characteristic	Patients, No. (%)		*P* value
	Linked and included in analysis (n = 1429)	Not linked or included in analysis (n = 1222)	
Age, median (range), y	71 (65-91)	71 (65-89)	.84
<75	970 (67.9)	825 (67.5)	
≥75	459 (32.1)	397 (32.5)	
Race			
Black	109 (7.6)	104 (8.5)	.38
White	1280 (89.6)	1075 (88.0)	
Other or unknown^a^	40 (2.8)	43 (3.5)	
Ethnicity			
Hispanic	38 (2.7)	46 (3.8)	.11
Not Hispanic	1391 (97.3)	1176 (96.2)	
Sex			
Female	306 (21.4)	281 (23.0)	.33
Male	1123 (78.6)	941 (77.0)	
Insurance status			
Medicare alone	352 (24.6)	297 (24.3)	<.001
Medicare and Medicaid	45 (3.1)	28 (2.3)	
Medicare and private	908 (63.5)	680 (55.6)	
Other	124 (8.7)	217 (17.8)	
Geographic location			
Rural	332 (23.5)	177 (14.9)	<.001
Urban	1078 (76.5)	1014 (85.1)	
Missing or unknown	19	31	
Area Deprivation Index			
Tertile 1	524 (37.6)	538 (45.7)	<.001
Tertile 2	513 (36.8)	418 (35.5)	
Tertile 3	356 (25.6)	222 (18.8)	
Missing or unknown	36	44	
Clinical data			
Prognostic risk score			
At or below median	1122 (78.5)	962 (78.7)	.90
Above median	307 (21.5)	260 (21.3)	
Type of cancer			
Pancreatic	457 (32.0)	543 (44.4)	<.001
Prostate	255 (17.8)	108 (8.8)	
Lung	717 (50.2)	571 (46.7)	
Performance status			
0-1	1391 (97.3)	1198 (98.0)	.24
≥2	38 (2.7)	24 (2.0)	

^a^ Other race category includes Asian, American Indian or Alaska Native, Pacific Islander, multiracial, and unknown.

**Figure 1.**
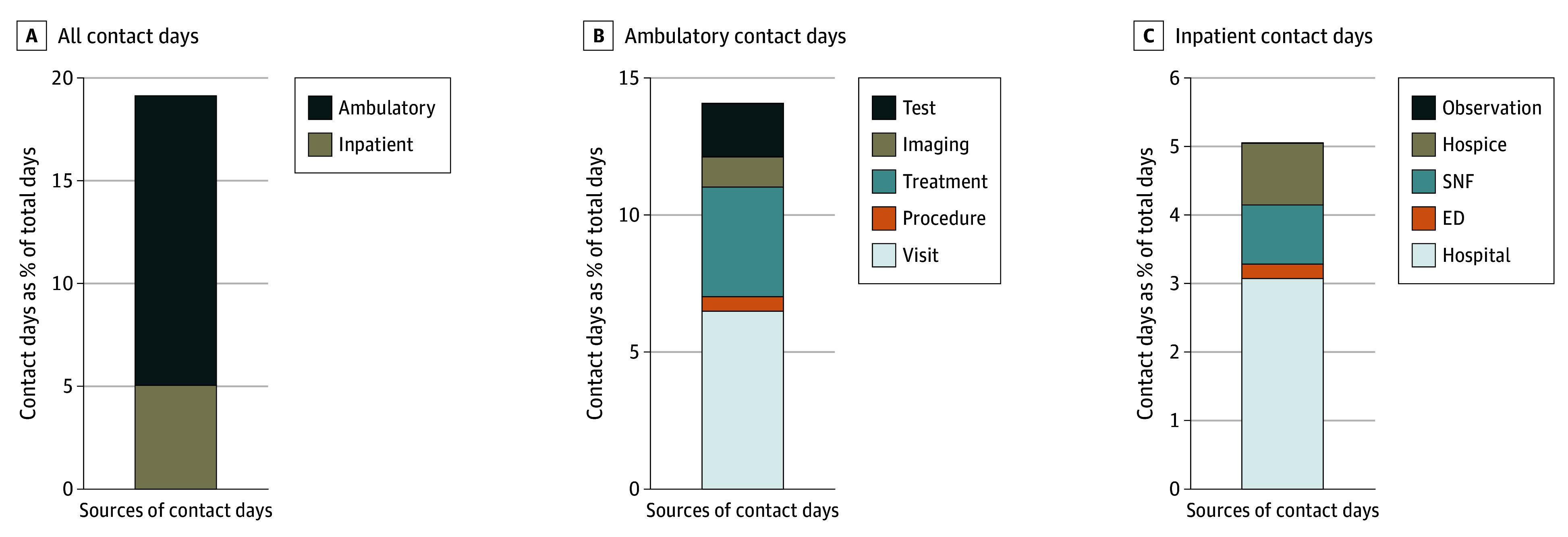
Components of Health Care Contact Days Among Clinical Trial Participants These stacked bar charts present the percentage of days with specific components of health care contact. A, Ambulatory vs inpatient care, following the hierarchy: inpatient care and then ambulatory care if both occur on the same day. B, Components of ambulatory contact days, following the hierarchy if more than 1 ambulatory component on the same day: visit then procedure then treatment and then imaging then test. C, Components of inpatient contact days, following the hierarchy if more than 1 inpatient component on the same day: inpatient care then emergency department (ED) then skilled nursing facility (SNF), and then hospice.

Overall, 1426 patients (99.8%) had some health care contact within the first 12 months after registration (Table 2); 741 patients (51.9%) died in the first year. The median number of contact days was 48 (IQR, 26-71) for the whole cohort, of a median of 350 total days (IQR, 178-365 total days) of observation. The median contact as a percentage of total days’ observation was 19% (IQR, 13%-29%). The median contact day rate ranged from 14% (IQR, 9%-21%) for prostate cancer to 27% (IQR, 18%-37%) for lung cancer across trials. Overall, patients experienced a median number of 6 contact days (IQR, 4-9 contact days) per month; the median number of contact days per month ranged from 4 to 8 across studies. In the S0003 trial, the actual number of contact days (median, 6 days per month) was more than double the expected number of contact days (2-3 days per month) (eTable 5 in Supplement 1). The most common sources of contact days were ambulatory clinician visits (median, 17 [IQR, 7-25]), tests (median, 12 [IQR, 3-24]), treatments (median, 11 [IQR, 3-22]), and imaging (median, 5 [IQR, 2-8]). Figure 1 presents the distribution of the components of contact days. More than two-thirds (median, 70% [IQR, 50%-88%]) of ambulatory contact days had only 1 service delivered on that day, and only 14% (IQR, 2%-31%) of ambulatory days with a clinician visit included another ambulatory service on the same day. The median 1-way travel distance for study-related care was 47 km (IQR, 15-230 km).

**Table 2.** Summary Statistics of Health Care Utilization in the First Year After Registration, Overall and by Study

Characteristic	Median (IQR)						
	All (N = 1429)	S0003 (lung cancer) (n = 118)	S0124 (lung cancer) (n = 180)	S0205 (pancreatic cancer) (n = 255)	S0421 (prostate cancer) (n = 375)	S0819 (lung cancer) (n = 159)	S9916 (prostate cancer) (n = 342)
Any contact, No. (%)	1426 (99.8)	118 (100.0)	180 (100.0)	255 (100.0)	374 (99.7)	158 (99.4)	341 (99.7)
Deaths, No. (%)	741 (51.9)	81 (68.6)	127 (70.6)	211 (82.7)	123 (32.8)	86 (54.1)	113 (33.0)
Duration of observation, median (IQR), d	350 (178-365)	233 (147-365)	256 (158-365)	175 (89-308)	365 (310-365)	312 (136-365)	365 (302-365)
Total contact days, No.	73 726	5981	10 576	11 704	20 282	9280	15 903
Total contact days, median (IQR)	48 (26-71)	48 (30-71)	57 (33-79)	42 (21-68)	47 (30-71)	58 (29-81)	44 (25-62)
Contact as a % of total days, median (IQR)	19 (13-29)	21 (15-34)	27 (18-37)	25 (18-35)	16 (10-24)	25 (16-33)	14 (9-21)
Contact days per month on trial, median (IQR)	6 (4-9)	6 (5-10)	8 (6-11)	8 (5-11)	5 (3-7)	8 (5-10)	4 (3-6)
Travel distance, median (IQR), km^a^	47 (15-230)	35 (11-103)	21 (8-77)	23 (10-71)	55 (18-460)	100 (28-573)	68 (18-480)
Contact days by category, median (IQR)							
Any inpatient care	6 (1-17)	10 (2-27)	11 (4-20)	8 (2-20)	4 (0-14)	7 (1-18)	3 (0-11)
Hospital	4 (0-12)	5 (0-15)	9 (2-16)	6 (1-12)	3 (0-10)	6 (0-13)	2 (0-9)
ED	0 (0-1)	0 (0-1)	0 (0-1)	0 (0-1)	0 (0-1)	0 (0-1)	0 (0-1)
SNF	0 (0-0)	0 (0-0)	0 (0-0)	0 (0-0)	0 (0-0)	0 (0-0)	0 (0-0)
Hospice	0 (0-0)	0 (0-0)	0 (0-0)	0 (0-0)	0 (0-0)	0 (0-0)	0 (0-0)
Observation	0 (0-0)	0 (0-0)	0 (0-0)	0 (0-0)	0 (0-0)	0 (0-0)	0 (0-0)
Any ambulatory care	36 (16-56)	32 (8-50)	39 (16-65)	27 (12-46)	39 (25-57)	45 (17-66)	36 (17-53)
Clinician visits	17 (7-25)	15 (3-22)	16 (5-23)	12 (4-21)	19 (11-26)	17 (7-28)	18 (9-26)
Tests	12 (3-24)	5 (2-18)	8 (2-21)	7 (2-15)	20 (8-28)	16 (5-31)	10 (3-21)
Imaging	5 (2-8)	5 (2-9)	6 (2-9)	4 (1-7)	5 (3-8)	8 (2-11)	5 (2-7)
Procedures	1 (0-2)	0 (0-1)	0 (0-2)	1 (0-2)	1 (0-3)	1 (0-3)	0 (0-2)
Treatments	11 (3-22)	8 (1-20)	17 (4-35)	10 (3-19)	14 (5-22)	17 (6-34)	7 (1-16)
% of Ambulatory days with only 1 service, median (IQR)^b^	70 (50-88)	77 (56-91)	78 (58-91)	75 (46-90)	61 (45-75)	58 (42-75)	79 (58-94)
% of Ambulatory days with ambulatory service on same day as a clinician visit, median (IQR)^c^	14 (2-31)	10 (0-27)	10 (0-20)	8 (0-25)	26 (11-39)	20 (9-32)	9 (0-27)

Abbreviations: ED, emergency department; SNF, skilled nursing facility.

^a^ One-way travel distance from residence zip code to study site zip code.

^b^ Includes clinician visits, tests, imaging, procedures, and treatments.

^c^ Includes tests, imaging, procedures, and treatments.

In multivariate regression, factors independently associated with contact days were age (RR, 1.02 [95% CI, 1.01-1.02]; *P* < .001), insurance status (RR, 2.47 [95% CI, 2.16-2.83] for Medicare alone or with Medicaid or private insurance compared with other insurance; *P* < .001), high prognostic risk (RR, 1.14 [95% CI, 1.04-1.25]; *P* = .004), and type of cancer (RR, 1.69 [95% CI, 1.51-1.89] for pancreatic compared with prostate cancer; *P* < .001; RR, 1.69 [95% CI, 1.54-1.85] for lung compared with prostate cancer; *P* < .001) **(**Table 3**)**. Results were similar when patients with no health care utilization were excluded and when considering only inpatient contact days (eTable 4 in Supplement 1).

**Table 3.** Associations Between Variables and Days of Contact With the Health Care System

Demographic data	No. (%) (N = 1429)	Contact days, mean (SD)	Univariate analyses		All variables mutually adjusted for each other	
			RR (95% CI)	*P* value	RR (95% CI)	*P* value
Age, per year	NA	NA	1.01 (1.00-1.01)	.09	1.02 (1.01-1.02)	<.001
Race						
Black	109 (7.6)	52.2 (36.6)	0.90 (0.78-1.04)	.16	0.98 (0.85-1.13)	.77
White	1280 (89.6)	52.0 (34.6)	1 [Reference]		1 [Reference]	
Other or unknown^a^	40 (2.8)	42.5 (30.5)	0.79 (0.63-0.99)	.04	0.97 (0.78-1.21)	.81
Ethnicity						
Hispanic	38 (2.7)	47.7 (34.2)	0.81 (0.64-1.02)	.07	0.98 (0.79-1.22)	.86
Not Hispanic	1391 (97.3)	51.8 (34.7)	1 [Reference]		1 [Reference]	
Sex						
Female	306 (21.4)	53.0 (32.2)	1 [Reference]		1 [Reference]	
Male	1123 (78.6)	51.4 (35.3)	0.77 (0.70-0.84)	<.001	1.02 (0.92-1.13)	.68
Insurance status						
Other	124 (8.7)	19.4 (23.8)	1 [Reference]		1 [Reference]	
Medicare alone or with Medicaid or private insurance	1305 (91.3)	54.7 (34.0)	2.36 (2.06-2.71)	<.001	2.47 (2.16-2.83)	<.001
Geographic location						
Rural	332 (23.5)	50.0 (33.7)	1 [Reference]		1 [Reference]	
Urban	1078 (76.5)	52.4 (35.0)	1.09 (1.00-1.19)	.06	1.05 (0.96-1.15)	.26
Area Deprivation Index						
Tertile 1	524 (37.6)	53.0 (34.2)	1 [Reference]		1 [Reference]	
Tertile 2	513 (36.8)	51.6 (36.3)	1.03 (0.94-1.13)	.53	1.00 (0.92-1.09)	.95
Tertile 3	356 (25.6)	51.1 (33.5)	0.96 (0.87-1.06)	.45	0.95 (0.86-1.05)	.32
Clinical data						
Prognostic risk score						
At or below median	1122 (78.5)	53.1 (34.7)	1 [Reference]		1 [Reference]	
Above median	307 (21.5)	46.8 (34.0)	1.08 (0.99-1.19)	.09	1.14 (1.04-1.25)	.004
Type of cancer						
Prostate	717 (50.2)	50.6 (35.7)	1 [Reference]		1 [Reference]	
Pancreas	255 (17.8)	45.9 (30.9)	1.59 (1.43-1.75)	<.001	1.69 (1.51-1.89)	<.001
Lung	457 (32.0)	56.7 (34.3)	1.62 (1.49-1.75)	<.001	1.69 (1.54-1.85)	<.001
Performance status						
0-1	1391 (97.3)	51.8 (34.8)	1 [Reference]		1 [Reference]	
≥2	38 (2.7)	47.3 (30.7)	0.91 (0.72-1.15)	.43	1.15 (0.91-1.46)	.23

Abbreviation: RR, relative risk.

^a^ Other race category includes Asian, American Indian or Alaska Native, Pacific Islander, multiracial, and unknown.

**Figure 2.**
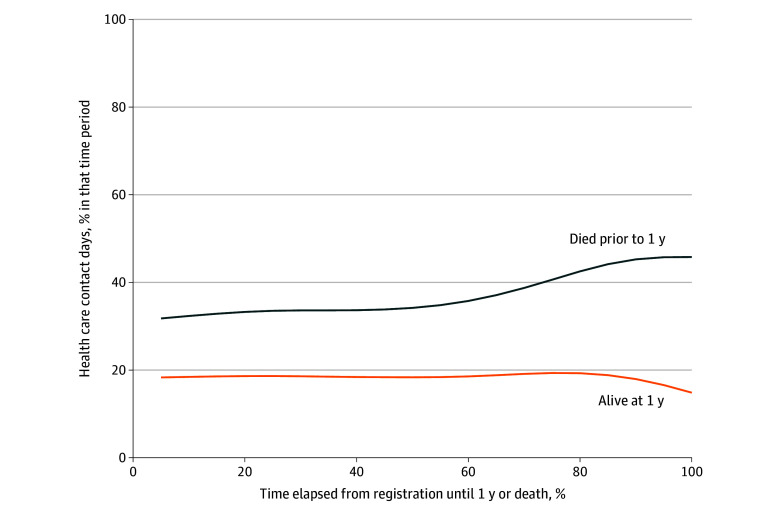
Trajectory of Percentage of Contact Days Over Normalized Follow-Up Period, Stratified by Patient Survival To ensure a fair comparison of contact day trajectories and facilitate visualization of contact day trajectories across patients with differential follow-up durations, irrespective of individual follow-up lengths, the time between trial enrollment and end of follow-up was split into 20 equal time periods per participant. The percentage of contact days in each time period for each participant was plotted. Cubic smoothing splines (lines) were fitted to estimate the trajectories of the time series observations.

For patients surviving less than 1 year (complete trajectories from enrollment to death captured), the contact day rate followed a flat trajectory at approximately 30% for the first half of the course, before increasing in the second half and peaking at just above 40% shortly before death (Figure 2). For patients surviving beyond 1 year (clinical course not completely captured in the 1-year follow-up period), the trajectory was flat throughout, at approximately 20%.

eTable 5 in Supplement 1 presents the expected number of protocol-mandated contact days. For the 6 trials or trial arms that delivered systemic therapy in 3-week cycles, the median expected number of contact days associated with systemic therapy receipt per cycle was 1, with an additional expected contact day every 2 to 4 cycles for imaging response assessment.

## Discussion

This study found that older adults with cancer participating in clinical trials spent nearly 1 in 5 days with health care contact. Ambulatory clinician appointments were the most common source of contact days, and 70% of ambulatory contact days involved delivering a single service (eg, only laboratory tests). Contact days varied largely by clinical characteristics. Rural residence and neighborhood deprivation were not associated with contact days, suggesting that care is not differently burdensome for socioeconomically disadvantaged patients within clinical trials. Trajectories of contact days were relatively flat, before an increase as patients approached the end of life. These findings highlight the frequency of contact days for older trial participants, identify factors to consider when benchmarking contact days, and offer improvement opportunities to minimize patient burden.

Prior work evaluating contact days in clinical trials have used alternative data sources: (1) clinical trial forms,^14,15,16^ (2) electronic medical records,^11,12^ and (3) trial protocols.^17^ These are limited by data quality (few trials collect detailed information on health care use), size (limited to single-center studies), and scope (accounting for only planned visits). Administrative claims data linked to trial data have demonstrated high accuracy when conducting economic analyses. Additional advantages include follow-up even after trial data collection ends and removal of onerous burdens and expenses from trial teams.^18^ Mobile health technologies (eg, geolocation services and sensor data) that passively record patient visits represent a valuable future way to collect data on contact days.^5^ For now, linking trial and claims data, even for a subset of trial populations, may be the most comprehensive method to analyze data on contact days at scale. This work establishes the feasibility of linking trial and claims data to evaluate contact days.

Analyzing contact days within the context of clinical trials represents a unique opportunity to evaluate burdens of care in a more homogenous population receiving more uniform care, which limits confounding by case mix and allows for the opportunity to address whether the burden of care in trials differs by participants’ sociodemographic backgrounds. Patients in this cohort had a median 19% contact day rate, and the number of contact days varied largely by clinical characteristics rather than by measures of neighborhood socioeconomic deprivation. These findings should be interpreted in the context of the underlying population and clinical trial care delivery. Across the included trials, the study population included older adults with advanced solid tumors who received cytotoxic chemotherapy with or without additional targeted therapy. Median survival was less than 12 months in 5 of 6 trials. This reflects an overall relatively sick population with high health care needs. The 19% contact day rate is lower than the 25% to 33% contact day rate observed for patients with advanced solid cancers receiving care in routine practice—a population that is even sicker than the population included in the included trials. In adjusted analysis, factors such as age (RR, 1.02 per year; a 5-year increase would represent 10% greater contact days) and the primary cancer type (pancreas and lung cancer vs prostate cancer) were associated with more contact days, further highlighting how clinical needs are likely associated with contact days.

Prior work has cautioned against the uniform interpretation of fewer contact days as universally good because, in some cases, contact days also represent access to necessary care.^13^ In prior studies assessing patterns of contact days in routine practice, patients belonging to racial and ethnic minority groups or residing in rural areas experienced fewer contact days, representing barriers to accessing care.^4,9^ In contrast, in the present study, race and ethnicity, rural residence, and neighborhood deprivation were not associated with contact days, implying that clinical trial participation may overcome care delivery disadvantages seen outside clinical trials. This may be because clinical trials tend to provide more protocol-guided care or because trial participation itself represents being able to surpass access barriers. Prior work has demonstrated that rural and urban trial participants with uniform access to cancer care had similar outcomes.^19^ However, commute time and the logistics of care can be very different for these populations. Alternative metrics to capture this part of the patient experience are still needed.^20,21^ We found opportunities for improving care: 70% of ambulatory contact days included only 1 service. Coordinating ambulatory appointments, especially for protocol-mandated visits, is a low-hanging target to address burdens. We also noted clear differences between optimal (as designed in trial protocols) and actual (as delivered) care. As an example, in the S0003 trial, the actual number of contact days (median, 6 days per month) was more than double the expected number of contact days (2-3 days per month).

The other notable finding of this study is the relatively uniform trajectory of the rate of contact days over time, especially in the first half of the clinical course. This contrasts with prior work in routine practice, which describes a U-shaped trajectory, highlighting an initial intensive phase (peridiagnosis, often with symptomatic needs and cancer workup), then a trough (during receipt of treatment, when things “settle”), and an increase toward the end (as the patient approaches the end of life).^8,9,10^ In the present study, while we did see an increase in contact days during the second half of the clinical course, the overall trajectory was different from the classic U shape replicated across multiple studies in routine practice. The flat trajectory in the first half (without the trough or decrease seen for patients treated in routine practice) may be because participants attended extra protocol-mandated visits, even when clinically well, thus preventing an expected decrease in rates of contact days. The eventual increase mirrored patterns seen in routine practice; thus, increasing contact days on clinical trial may be an early warning sign of impending sickness. Trajectory-based analysis thus represents a valuable tool to assess patterns of contact days. Future work can evaluate how patterns of time toxicity differ between trial participants and patients treated in routine practice.

### Limitations

This study has some limitations. First, these data do not reflect the experiences of all older adults enrolled in cancer clinical trials. Patterns may be different for those receiving oral targeted therapy or hormonal therapy. Second, we did not seek to adjudicate the clinical appropriateness of a contact day, or whether a day was protocol mandated. This is difficult to do retrospectively and without a detailed understanding of the clinic context, which is not routinely available in study case report forms. However, our separate sensitivity analysis of inpatient contact days (as a measure of non–protocol-related care) showed similar associations as total number of contact days. Additional multivariable analysis of disaggregated types of contact days was not conducted given the potential to add unnecessary complexity with limited benefit in terms of additional interpretation. We were also unable to specifically examine in the follow-up period if and when a clinical trial ended and routine care started. In addition, this study focused on characteristics at baseline and did not account for the potentially time-varying nature of the variables. Third, participants were recruited from 1999 to 2014, and studies were completed prior to 2020. Insurance coverage, such as the proliferation of Medicare Advantage, and care delivery paradigms, with increasing telemedicine and home-based care, have since evolved.

## Conclusions

This cohort study analysis of patterns of contact days for older clinical trial participants with advanced stage cancer provides important insights into overall patient burden (every fifth day with health care contact) and benchmarking contact days in clinical trials (consider primarily clinical factors), while identifying opportunities to improve trial design and decrease participant burden (decreasing unnecessary visits and coordinating outpatient appointments).

## Notes

Supplement 1. **eTable 1.** Prognostic Risk Score **eTable 2.** Contact Days Definitions by Category **eTable 3.** Inclusion Flow Diagram **eTable 4.** Sensitivity Analyses of the Associations Between Predictors and Days of Contact With the Healthcare System **eTable 5.** Expected Patterns of Contact Days Based on Study Protocols

Supplement 2.
Data Sharing Statement
