# Supplementary material for: Health Care Contact Days for Older Adults Enrolled in Cancer Clinical Trials
Source: JAMA Netw Open. 2025 Mar 13;8(3):e250778. doi: 10.1001/jamanetworkopen.2025.0778 (PMC11907310; doi:10.1001/jamanetworkopen.2025.0778)
Supplement: Supplement 1. — eTable 1. Prognostic Risk Score eTable 2. Contact Days Definitions by Category eTable 3. Inclusion Flow Diagram eTable 4. Sensitivity Analyses of the Associations Between Predictors and Days of Contact With the Healthcare System eTable 5. Expected Patterns of Contact Days Based on Study Protocols [file jamanetwopen-e250778-s001.pdf]

Supplementary Online Content

Gupta A, Till C, Vaidya R, Hershman DL, Unger JM. Health care contact days for older adults enrolled in cancer clinical trials. *JAMA Netw Open.* 2025;8(3):e250778. doi:10.1001/jamanetworkopen.2025.0778

eTable 1. Prognostic Risk Score
eTable 2. Contact Days Definitions by Category
eTable 3. Inclusion Flow Diagram
eTable 4. Sensitivity Analyses of the Associations Between Predictors and Days of Contact With the Healthcare System
eTable 5. Expected Patterns of Contact Days Based on Study Protocols

This supplementary material has been provided by the authors to give readers additional information about their work.

© 2025 Gupta A et al. *JAMA Network Open.*

eTable 1. Prognostic Risk Score

Study ID	Risk Factor	Risk score	N (%) with each factor	Definition of "high risk"	N (%) high risk
S0003 (Lung)	weight: <5%	0	84 (71.2%)	>1	58 (49.2%)
	weight: >=5%	1	34 (28.8%)		
	stage: IIIB	0	17 (14.4%)		
	stage: IV	1	101 (85.6%)		
	ldh: <=IULN	0	74 (62.7%)		
	ldh: >IULN	1	44 (37.3%)		
S0124 (Lung)	LDH: <= IULN	0	58 (32.2%)	>2	34 (18.9%)
	LDH: > IULN	1	122 (67.8%)		
	Metastatic Sites: Single	0	44 (24.4%)		
	Metastatic Sites: Multiple	1	136 (75.6%)		
	Weight Loss: <= 5%	0	110 (61.1%)		
	Weight Loss: > 5%	1	70 (38.9%)		
S0205 (Pancreas)	Disease status: Locally advanced unresectable	0	63 (24.7%)	>2	20 (7.8%)
	Disease status: Metastatic	1	192 (75.3%)		
	Performance status: 0 or 1	0	227 (89.0%)		
	Performance status: 2	1	28 (11.0%)		
	Prior pancreatectomy: Yes	0	24 (9.4%)		
	Prior pancreatectomy: No	1	231 (90.6%)		
S0421 (Prostate)	Type of prog: Meas/eval	0	301 (80.3%)	>2	66 (17.6%)
	Type of prog: PSA only	1	74 (19.7%)		
	Bisphos use: Yes	1	217 (57.9%)		
	Bisphos use: No	0	158 (42.1%)		
	Worst pain: < 4	0	235 (62.7%)		
	Worst pain: >= 4	1	140 (37.3%)		
	Extraskeletal mets: Yes	1	211 (56.3%)		
	Extraskeletal mets: No	0	164 (43.7%)		
S0819 (Lung)	BEV_STATUS: Bevacizumab-INAPPROPRIATE	1	91 (57.2%)	>2	19 (11.9%)
	BEV_STATUS: Bevacizumab-APPROPRIATE	0	68 (42.8%)		
	SMOKE_HX: Current	1	40 (25.2%)		
	SMOKE_HX: Former	0	106 (66.7%)		
	SMOKE_HX: Never	0	13 (8.2%)		
	M_STAGE: M1a	0	37 (23.3%)		
	M_STAGE: M1b	1	122 (76.7%)		
S9916 (Prostate)	Type of Prog: Meas/Eval	1	270 (78.9%)	>1	109 (32.2%)
	Type of Prog: PSA Only	0	72 (21.1%)		
	Bone Pain: Grade >= 2	1	118 (34.5%)		

© 2025 Gupta A et al. *JAMA Network Open.*

Study ID	Risk Factor	Risk score	N (%) with each factor	Definition of "high risk"	N (%) high risk
	Bone Pain: Grade < 2	0	224 (65.5%)		
	PS: 0-1	0	303 (88.6%)		
	PS: 2-3	1	39 (11.4%)		

© 2025 Gupta A et al. *JAMA Network Open.*

eTable 2. Contact Days Definitions by Category

**Inpatient, ED, and Facility-Based Care**	**Medicare Source Data Set**	**Specifics**	**Days of Contact**
Inpatient	Medicare Provider Analysis and Review (MedPAR)	NCH Claim Type Code 60-64, 71, 82 or if NCH Claim Type Code is missing, then SNF Indicator Code not 'N' or missing	Admission Date and Discharge Date, plus all days in between
ED Visit	MedPAR and Outpatient	Emergency Charge Amount non-missing and non-zero (MedPAR) or Revenue Center Code 0450-0459 or 0981 (Outpatient)	1 day for each unique Admission Date (MedPAR) or Claim Through Date (Outpatient)
SNF	MedPAR	SNF Indicator Code 'N'	Admission Date and Discharge Date, plus all days in between
Hospice	Hospice	Claim Service Classification Type Code 2	Claim From Date and Claim Through Date, plus all days in between
Observation Stay	Outpatient	Revenue Center Code 0762, 0760 and HCPCS 99218-99220, 99224-99226, G0378, G0379	1 day for each unique Claim Through Date
**Ambulatory Care**	**BETOS Codes in the Carrier File**	**HCPCS and Revenue Center Codes in the Outpatient File**	**Days of Contact**
Clinician Visit	BETOS Code M1A, M1B, M5B, M5C, M5D	HCPCS 99201-99205, 99211-99215, 99241-99245, 99386, 99387, 99396, 99397	1 day for each unique Claim Through Date
Tests	BETOS Codes T1A - T1H, T2A - T2D	Revenue Center Codes 030X, 031X, 046X, 048X, 073X, 074X, 092X, 0971	
Imaging	BETOS Codes I1A -I1F, I2A-I2D, I3A - I3F, I4A - I4B	Revenue Center Codes 032X, 035X, 040X, 061X, 086X, 0340, 0341, 0343, 0349	
Procedures	BETOS Codes O1B, P0, P1A - P1G, P2A - P2F, P3A - P3D, P4A - P4E, P5A - P5E, P6A - P6D, P8A - P8I	Revenue Center Codes 036X, 037X, 038X, 039X	
Treatments	BETOS Codes D1G, O1D, O1E, P7A - P7B, P9A - P9B	Revenue Center Codes 025X, 026X, 028X, 033X, 042X, 043X, 044X, 063X, 080X, 087X, 093X, 094X, 095X, 0294, 0342, 0344	

© 2025 Gupta A et al. *JAMA Network Open.*

eTable 3. Inclusion Flow Diagram

	S0003	S0124	S0205	S0421	S0819	S9916	All
Total registered	397	671	766	1038	1333	770	4975
Exclusions							
Age <65	231	405	403	324	765	196	2324
No match to Medicare or inadequate coverage	41	79	107	305	403	186	1121
No observed utilization (unless by reported protocol deviation)	7	7	1	34	6	46	101
**Final numbers in analysis**	**118**	**180**	**255**	**375**	**159**	**342**	**1429**

© 2025 Gupta A et al. *JAMA Network Open.*

eTable 4. Sensitivity Analyses of the Associations Between Predictors and Days of Contact With the Healthcare System

a) **excluding patients with no observed utilization**
b) **with only inpatient contact days as the outcome**

	**Removing people without utilization**				**Inpatient Contact Days Only**			
	**N (%)**	**Contact Days, Mean (SD)**	**Relative Risk (95% CI)**	**p-value**	**N (%)**	**Contact Days, Mean (SD)**	**Relative Risk (95% CI)**	**p-value**
**Demographic**								
Age			1.02 (1.01, 1.02)	<0.0001			1.05 (1.03, 1.07)	<0.0001
Race								
Black	108 (7.6%)	52.2 (36.6)	0.98 (0.85, 1.13)	0.77	109 (7.6%)	15.7 (23.1)	1.02 (0.70, 1.47)	0.93
White	1278 (89.6%)	51.9 (34.6)	Ref		1280 (89.6%)	13.6 (22.7)	Ref	
Other/Unknown[1]	40 (2.8%)	42.5 (30.5)	0.97 (0.78, 1.21)	0.81	40 (2.8%)	10.3 (14.1)	0.79 (0.44, 1.41)	0.42
Ethnicity								
Not Hispanic	1388 (97.3%)	51.8 (34.7)	Ref		1391 (97.3%)	13.6 (22.7)	Ref	
Hispanic	38 (2.7%)	47.7 (34.1)	0.98 (0.79, 1.22)	0.86	38 (2.7%)	13.9 (18.5)	1.21 (0.68, 2.14)	0.52
Sex								
Female	305 (21.4%)	53.0 (32.2)	Ref		306 (21.4%)	15.1 (19.9)	Ref	
Male	1121 (78.6%)	51.3 (35.3)	1.02 (0.92, 1.13)	0.68	1123 (78.6%)	13.3 (23.2)	1.06 (0.82, 1.38)	0.65
Insurance status								
Other	121 (8.5%)	19.4 (23.8)	Ref		124 (8.7%)	8.6 (13.7)	Ref	
Medicare Alone or with Medicaid or Private Insurance	1305 (91.5%)	54.7 (34.0)	2.47 (2.16, 2.83)	<0.0001	1305 (91.3%)	14.1 (23.2)	1.53 (1.08, 2.18)	0.02
Geographic location								
Rural	332 (23.6%)	49.9 (33.7)	Ref		332 (23.5%)	12.8 (22.1)	Ref	

© 2025 Gupta A et al. *JAMA Network Open.*

Urban	1075 (76.4%)	52.4 (35.0)	1.05 (0.96, 1.15)	0.26	1078 (76.5%)	14.0 (22.8)	1.13 (0.89, 1.44)	0.30
ADI								
ADI T1	521 (37.5%)	53.0 (34.2)	Ref		524 (37.6%)	13.6 (21.5)	Ref	
ADI T2	513 (36.9%)	51.5 (36.3)	1.00 (0.92, 1.09)	0.95	513 (36.8%)	13.0 (24.9)	1.04 (0.83, 1.29)	0.75
ADI T3	356 (25.6%)	51.1 (33.5)	0.95 (0.86, 1.05)	0.32	356 (25.6%)	14.9 (21.3)	1.04 (0.80, 1.34)	0.79
**Clinical Data**								
Prognostic risk score								
Below median	1121 (78.6%)	53.0 (34.7)	Ref		1122 (78.5%)	13.3 (22.7)	Ref	
Above median	305 (21.4%)	46.8 (34.0)	1.14 (1.04, 1.25)	0.004	307 (21.5%)	15.0 (21.8)	1.56 (1.23, 1.99)	<0.001
Type of cancer								
Prostate	715 (50.1%)	50.6 (35.7)	Ref		717 (50.2%)	11.9 (24.6)	Ref	
Pancreas	255 (17.9%)	45.9 (30.9)	1.69 (1.51, 1.89)	<0.0001	255 (17.8%)	14.9 (20.3)	2.90 (2.17, 3.89)	<0.0001
Lung	456 (32.0%)	56.7 (34.3)	1.69 (1.54, 1.85)	<0.0001	457 (32.0%)	15.7 (20.1)	2.63 (2.07, 3.35)	<0.0001
Performance Status								
0-1	1389 (97.4%)	51.8 (34.8)	Ref		1391 (97.3%)	13.6 (22.7)	Ref	
2+	37 (2.6%)	47.3 (30.7)	1.15 (0.91, 1.46)	0.23	38 (2.7%)	16.0 (18.5)	1.28 (0.69, 2.36)	0.43

[1] Other race category includes Native American, Pacific Island, Asian, Multiracial, and Unknown

© 2025 Gupta A et al. *JAMA Network Open.*

eTable 5. Expected Patterns of Contact Days Based on Study Protocols[a]

Trial	Systemic therapy cycle	Expected contact days for receipt of systemic therapy	Imaging response assessment period	Expected contact days for imaging assessment	Additional expected contact days due to trial-related procedures [b]
**S0003**	3 weeks	1	6 weeks	1	0
**S0124**	4 weeks	3	8 weeks	1	0
	3 weeks	3	6 weeks	1	0
**S0205**	4 weeks	4	8 weeks	1	0
**S0421**	3 weeks	1	12 weeks	1	0
**S0819**	3 weeks	1	6 weeks	1	0
	3 weeks	3	6 weeks	1	0
**S9916**	3 weeks	1	9 weeks	1	0

[a] Trials may have more than 1 schedule due to differences in patterns between study arms

[b] Although all trials included additional requirements, for example, administration of oral chemotherapy, laboratory assessments, and toxicity checks, they would not necessarily cause additional contact days beyond those expected with delivering parenteral systemic therapy and imaging response assessments (e.g., complete blood count prior to chemotherapy would be expected to be on the same day).

© 2025 Gupta A et al. *JAMA Network Open.*
