# Supplementary material for: Health Care Contact Days for Older Adults Enrolled in Cancer Clinical Trials
Source: JAMA Netw Open. 2025 Mar 13;8(3):e250778. doi: 10.1001/jamanetworkopen.2025.0778 (PMC11907310; doi:10.1001/jamanetworkopen.2025.0778)
Supplement: Supplement 2. — Data Sharing Statement [file jamanetwopen-e250778-s002.pdf]

Data Sharing Statement

Gupta. Health Care Contact Days for Older Adults Enrolled in Cancer Clinical Trials. *JAMA Netw Open*. Published March 13, 2025. doi:10.1001/jamanetworkopen.2025.0778

Data
Data available: Yes
Data types: Deidentified participant data
How to access data: Email corresponding author.
When available: With publication

Supporting Documents
Document types: None

Additional Information
Who can access the data: Upon reasonable request.
Types of analyses: Specified purpose.
Mechanisms of data availability: After approval.
Any additional restrictions: None.
